# High quality clinical grade human embryonic stem cell lines derived from fresh discarded embryos

**DOI:** 10.1186/s13287-017-0561-y

**Published:** 2017-06-05

**Authors:** Jinpei Ye, Nicola Bates, Despina Soteriou, Lisa Grady, Clare Edmond, Alex Ross, Alan Kerby, Philip A. Lewis, Tope Adeniyi, Ronnie Wright, Kay V. Poulton, Marcus Lowe, Susan J. Kimber, Daniel R. Brison

**Affiliations:** 1 Division of Cell Matrix Biology and Regenerative Medicine, School of Biology, Faculty of Biology, Medicine and Health, University of Manchester, Manchester Academic Health Sciences Centre, 46 Grafton Street, Manchester, M13 9NT UK; 2 Department of Reproductive Medicine, Old St Mary’s Hospital, Central Manchester University Hospitals NHS Foundation Trust, Manchester Academic Health Sciences Centre, Oxford Road, Manchester, M13 9PW UK; 3 Genomic Diagnostics Laboratory, Manchester Centre for Genomic Medicine, Saint Mary’s Hospital, Central Manchester University Hospitals NHS Foundation Trust, Manchester Academic Health Sciences Centre, Oxford Rd, Manchester, M13 9WL UK; 4 Transplantation Laboratory, Manchester Royal Infirmary, Central Manchester University Hospitals NHS Foundation Trust, Manchester Academic Health Sciences Centre, 46 Grafton Street, Manchester, M13 9NT UK; 5 Maternal and Fetal Health Research Centre, Division of Developmental Biology & Medicine, School of Medicine, Faculty of Biology, Medicine and Health, University of Manchester, Manchester Academic Health Sciences Centre, 46 Grafton Street, Manchester, M13 9NT UK; 6 North West Embryonic Stem Cell Centre, Central Manchester University Hospitals NHS Foundation Trust and University of Manchester, Oxford Road, Manchester, M13 9WL UK; 7 Present Address: Institute of Biomedical Sciences, Shanxi University, Taiyuan, China

**Keywords:** Embryo, Human embryonic stem cells, Good Manufacturing Practice, Pluripotency

## Abstract

**Background:**

Human embryonic stem cells (hESCs) hold tremendous promise for cell replacement therapies for a range of degenerative diseases. In order to provide cost-effective treatments affordable by public health systems, HLA-matched allogeneic tissue banks of the highest quality clinical-grade hESCs will be required. However only a small number of existing hESC lines are suitable for clinical use; they are limited by moral and ethical concerns and none of them apply Good Manufacturing Practice (GMP) standards to the earliest and critical stages of gamete and embryo procurement. We thus aimed to derive new clinical grade hESC lines of highest quality from fresh surplus GMP grade human embryos.

**Methods:**

A comprehensive screen was performed for suitable combinations of culture media with supporting feeder cells or feeder-free matrix, at different stages, to support expansion of the inner cell mass and to establish new hESC lines.

**Results:**

We developed a novel two-step and sequential media system of clinical-grade hESC derivation and successfully generated seven new hESC lines of widely varying HLA type, carefully screened for genetic health, from human embryos donated under the highest ethical and moral standards under an integrated GMP system which extends from hESC banking all the way back to gamete and embryo procurement.

**Conclusions:**

The present study, for the first time, reports the successful derivation of highest-quality clinical-grade hESC lines from fresh poor-quality surplus human embryos generated in a GMP-grade IVF laboratory. The availability of hESC lines of this status represents an important step towards more widespread application of regenerative medicine therapies.

**Electronic supplementary material:**

The online version of this article (doi:10.1186/s13287-017-0561-y) contains supplementary material, which is available to authorized users.

## Background

The advent of the first derivation of embryonic stem cells in the mouse [1] and human [2] has stimulated tremendous interest in the study and application of these cells, which are naturally pluripotent and have the ability to differentiate into any cell type in the body after practically unlimited self-renewal in the stem cell state. Human embryonic stem cells (hESCs) hold enormous promise as tools for understanding normal human development and diseases and for new drug discovery. Perhaps the most important potential application of hESCs is the generation of cells and tissues that could be used for cell-based therapies for devastating and currently incurable disorders, such as spinal cord injury, neurological disease and blindness. Given an unlimited supply of undifferentiated cells, it is likely that any major technical obstacles to the generation of hESC-differentiated somatic lineages required for cell therapy will be overcome [3]. However, in order to provide cells with defined quality characteristics that are safe for the patient, Good Manufacturing Practice (GMP) needs to be employed [4]. Currently more than a thousand hESC lines have been derived and registered worldwide, but the vast majority are suitable only for research purposes due to the source of embryonic material, derivation process and subsequent handling procedures [5, 6]. There are specific requirements for hESC lines intended for clinical use. Such hESCs should be derived under stringent ethical guidelines, from traceable and tested donors, in a GMP-grade culture and quality management system. GMP [4] is defined by both the European Medicines Agency and the US Food and Drug Administration, in order to meet a number of pre-set specifications with regard to the development, manufacturing, and quality control of medicinal products aimed for clinical use. Although it is possible to apply GMP to animal components by careful risk assessment in certain circumstance (if no better options are available), an animal (xeno)-free and ultimately chemically defined culture system is preferable. Legislation is continuously being updated to keep pace with the progress in regenerative medicine, setting out more specific technical requirements and new standards for clinical safety development for advanced medicinal products [7]. Therefore a detailed strategy has to be defined with regard to the goals for all components involved in the production of hESCs to meet current GMP.

Modifications and improvements have been repeatedly achieved for specific components of hESC derivation in order to obtain high-quality hESC lines for clinical use, including inner cell mass (ICM) isolation, development of human feeder cells or feeder-free substrates and fully chemically defined culture media devoid of animal products [8], etc. (for review, see [9–11]). However, despite great effort worldwide over the past nearly two decades, only a small number of clinical-grade hESC lines have been derived and relatively recently [3, 12–15]. Notably the first GMP-grade hESC lines derived *ab initio *were reported in 2007 [16] made in Singapore from embryos imported from Australia. However, animal products were used in the derivation system. Considerable progress has been made to obtain xeno-free GMP-grade hESCs in recent years [13, 17, 18]; but the gametes have not been sourced nor embryos generated in GMP-compliant laboratories. Moreover, so far the reported clinical-grade hESCs were all derived from donated high-quality embryos that were surplus to use in assisted reproduction. De Sousa and colleagues reported very recently on eight clinical-grade hESC lines derived from both high-quality frozen embryos and fresh discarded/failed-to-fertilise embryos, however, the quality of embryos was not shown [13]. Moreover, none of those lines was established as xeno-free: the initial derivation step at embryo plating and growth of ICM was xeno-free, but the expanding of nascent colonies to form a line was not under xeno-free conditions [13, 19, 20].

There is a clear need for more clinical-grade hESC lines in order to provide tissue banks of sufficient coverage for provision of allogeneic cell replacement therapies. After an approximately 10-year lag period since hESCs were first reported in 1998 [2], their use in clinical therapies has recently increased dramatically, with a number of trials in spinal cord injury, blindness, diabetes and heart failure [3, 21]. However, hESC lines are known to vary in their propensity to generate specific cell lineages [22] exemplified by our own data for hESC chondrogenesis [23]. The clinical utility and application of hESC lines is also limited by histocompatibility and immunological tolerance of the host. This issue can be accommodated by generating sufficient numbers of lines with a wide coverage of major histocompatibility complex haplotypes, increasing the likelihood that human leucocyte antigen (HLA) matching can be achieved where necessary [24]. Careful scrutiny of genetic health is also required, extending beyond karyotype to high-resolution investigation of hESC lines and their cell products for clinical use.

The safest option to obtain clinical-grade cells is to derive the hESC lines from the very beginning in GMP conditions, including the critical in vitro fertilisation (IVF) laboratory processes by which the gametes are procured, and the embryo generated and then procured for stem cell derivation. However, previous clinical-grade lines have been derived exclusively from non-GMP-grade embryos and the majority of them were from cryopreserved and well-developed embryos, originally stored for the patients’ own use in fertility treatment and subsequently donated to research. There are two major disadvantages of such lines; the first concerns the source and handling of the embryonic starting material in IVF laboratories operating at non-European Union Tissues and Cells Directive (EUTCD)/GMP standards. Second, there are ethical and moral concerns over the use of cryopreserved embryos which have been stored for the treatment of the parent couple or could be donated to (“adopted by”) another infertile couple. In order to minimise this ethical concern, we consider that it is important to derive hESC lines from oocytes or fresh IVF embryos which cannot be used for clinical treatment [25–27].

Therefore, for both safety and ethical reasons, the next significant advance towards clinical use of hESC lines is to derive them from embryos created in an IVF laboratory designed and operated to GMP standards but which are then surplus to treatment at the point of patient care, under complete xeno-free and GMP conditions. However, as these embryos have very limited developmental potential, this advance also requires the development of a novel and robust protocol in order to derive stem cell lines efficiently from such material.

Here we report the derivation and characterisation of the most advanced clinical-grade hESC lines with the highest ethical and moral status to date. We developed a highly efficient clinical-grade hESC derivation culture system without expensive specialist micromanipulation equipment, from fresh, discarded and poor-quality embryos that are not suitable for patient treatment under stringent GMP and xeno-free conditions from the point of gamete procurement in the IVF laboratory onwards. This development brings the execution of pre-clinical and clinical trials using pluripotent stem cells in regenerative medicine an important step closer.

## Methods

### GMP source and handling of fresh discarded human embryos

All work with human embryos and hESC derivation was carried out with approval of Central Manchester Local Research Ethics Committee (03/CM/684) and the Human Fertilisation and Embryology Authority (HFEA) licence R0067 and R0171. Informed consent was obtained from all couples who donated their embryos for the present study. As standard, all donating couples were screened for transmissible diseases [13], and starting gametes were procured and embryos produced in the clinical IVF laboratories at St Mary’s Hospital in Manchester, which were designed, built and operated at GMP standards and in compliance with the HFEA and the European Union Tissue and Cells Directive (Directives 2004/23/EC and 2006/17/EC). All the procedures in handling, culturing and manipulating human embryos were carried out in accordance with the HFEA Code of Practice (7th edition). The derivation of clinical-grade lines was carried out in our clinical-grade facility in the North West Embryonic Stem Cell Centre (NWESCC) under a GMP Quality Management System which is covered by the HFEA licence R0171, a licence for clinical application from the Human Tissue Authority (HTA; Licence 22627), a Certificate of GMP compliance and a Product Manufacturing Licence from the Medicines and Healthcare products Regulatory Agency (MHRA). Only embryos which were discarded by the team of clinical embryologists were collected for further culture in the present study in a system described in the Additional file 1.

### Derivation of hESC lines

After mechanical and manual disruption of the trophoblast cells or as a whole, the embryos were plated on different adhesive matrices and GMP-qualified human dermal fibroblast (hDF) cells inactivated by treatment with mitomycin C in various culture media. The primary colonies of hESCs were passaged by manual dissection up to passage 8, from which point the cells were then robustly passaged with Trypzean to establish the lines. Details regarding manipulation of embryos and primary colonies of hESCs and culture media/matrices are provided in the Additional file 1.

### Characterisation of hESC lines

All hESC lines were characterised using in-process and final characterisation methods, including immunocytochemistry evaluating the expression of pluripotent stem cells markers, karyotype with G-banding, comparative genomic hybridisation (CGH) arrays, in vitro/vivo differentiation with embryoid body and teratoma formation, and HLA typing, as described in Additional file 1.

Detailed procedures are provided in Additional file 1.

## Results

### Development of a xeno-free hESC derivation system at GMP

One of the major challenges in the development of a derivation system for clinical-grade hESC lines has been the lack of a suitable combination of culture medium with supporting feeder cells or feeder-free matrix to support expansion of the inner cell mass (ICM) [28]. We initially used human placental stromal fibroblasts (hPSFs) to replace mouse embryonic fibroblasts (MEFs) as feeder cells with xeno-free knock-out serum (KO-SR ZF) hESC medium [29]. However, hPSFs had to be utilised before passage 4 to establish lines successfully, and we were unable to generate a suitable GMP-grade hPSF line (data not shown). In order to provide consistent and reproducible derivation conditions we therefore chose a commercial GMP source of human dermal fibroblasts (hDFs) and propagated them in our GMP facility under xeno-free conditions. In preliminary experiments, we grew established hESC lines (Hues1 and Hues7) for three passages on four different GMP hDF feeder cell lines, selecting two of these lines (p107080110 and p106090049) based on ability to support hESC self-renewal and pluripotency (data not shown).

We next performed a screen for GMP-compatible culture media suitable for the derivation of hESCs with hDFs as feeders. We compared each culture system (combination of culture medium with hDFs) for long-term expansion of existing hESC lines. The culture media we tested included KO-SR ZF, NutriStem, StemPro, hESF9, TeSR2, Advanced (modified from [30]). Although some colony formation was obtained in all of these media, only TeSR2 and hESF9 gave robust growth of high-quality colonies. However, when we attempted to derive incipient new lines and then passage these on, none of the media alone could support a long-term culture of hESC lines on hDFs beyond passage 3. Accordingly no hESC line was established on hDFs with any single-stage medium.

#### Development of derivation protocol

Medium hESF9 was able to support hESC proliferation for more than four passages on hDFs, although the cells showed some tendency to undergo differentiation. However, we started by attempting derivations in hESF9. The seeded ICM attached on the feeders and initiated growth with proliferation to form a “dome-like” structure, but the growth resulted in rapid differentiation early during derivation. Different culture conditions were therefore employed immediately after the first two passages of the primary ICM-derived cell colony leading to a successful derivation. A part of the colony outgrowth was transferred onto MEFs in standard hESC medium (KO-SR) and gave rise to a hESC line (MAN9 m), while another part of the same outgrowth was transferred onto new hDFs in medium TeSR2 and gave rise to a research-grade line, MAN9. In contrast, the part which remained from this outgrowth continued to be passaged on the hDFs in medium hESF9, but differentiated significantly losing all colony-forming cells after several passages. The above successful procedure coupled with TeSR2 medium for the establishment of a line is entirely GMP-compatible using a two-step culture system on hDFs.

**Fig. 1:**
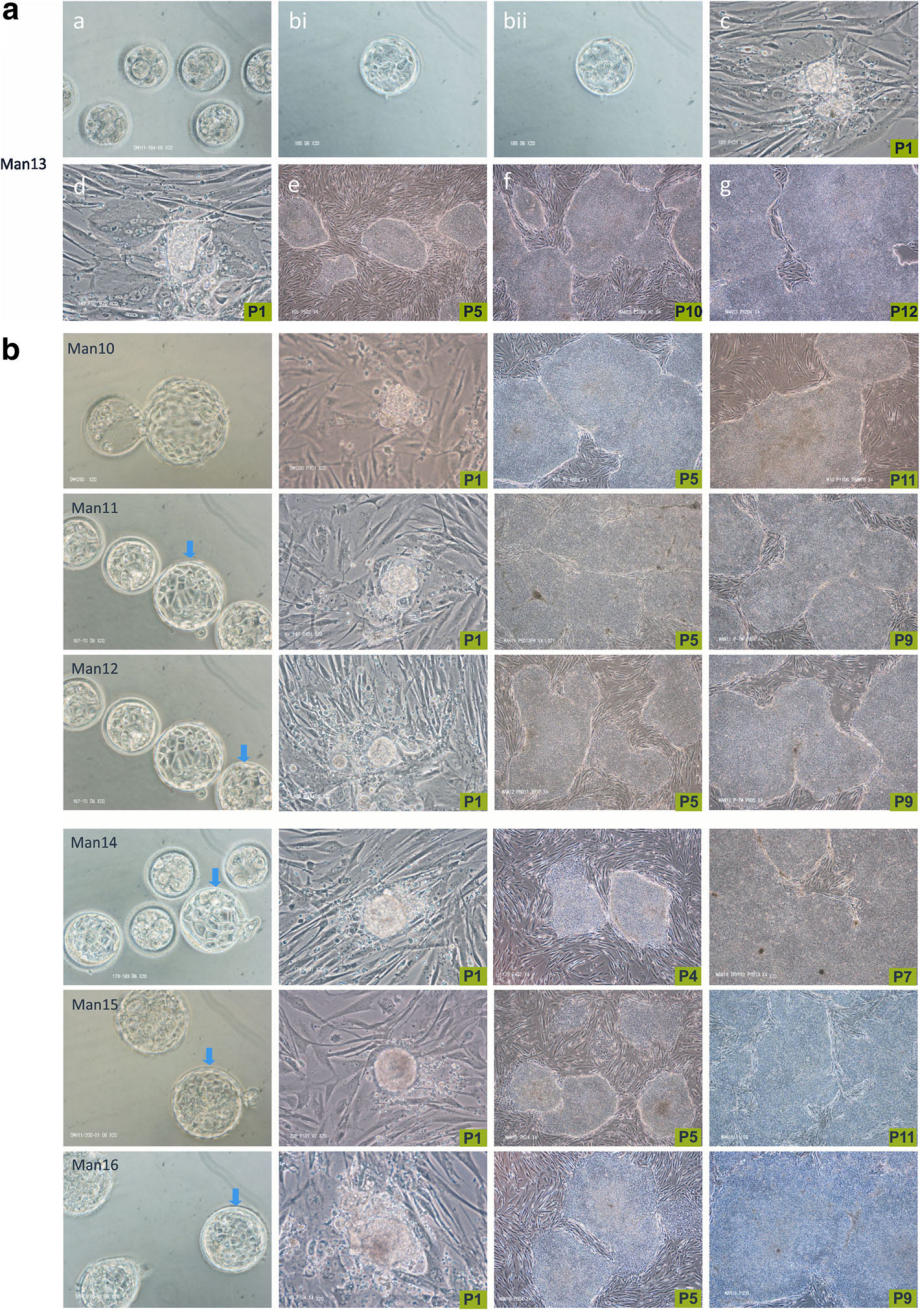
**a** Derivation of human embryonic stem cell lines MAN lines 10–16 from surplus fresh IVF embryos. The derivation of human embryonic stem cell line MAN13 from (a) surplus fresh IVF embryos donated on day 4 post-fertilisation, which gave rise to (b) a blastocyst on day 6 of grade BL3Dc, with no discernible inner cell mass (two different focal planes through the blastocyst, bi and bii). Following plating onto hDF feeder cells, an outgrowth was established (c) and passaged (passage 1, P1) (d) in order to establish MAN13 at P5 – P12 (e- g), which could be maintained on a different line of hDF feeder cells (f). **b** The derivation of human embryonic stem cell lines MAN10–12 and MAN 14–16 on hDF feeder cells. *Arrows* indicate blastocysts used in derivation

To confirm that it was possible to derive GMP-grade hESC lines with this novel two-step medium system using sequentially hESF9 and TeSR2 on hDFs, further derivations were performed in our clean room, resulting in the lines MAN10–14. MAN10 onwards were derived in the GMP-grade clean room, and MAN11-16 under an HTA licence for clinical application (22627). This GMP derivation system for clinical-grade hESC lines employing clinical-grade hDF feeder cells and the sequential culture media hESF9 followed by TeSR2 is highly efficient: lines were derived from blastocysts with almost invisible ICMs e.g. for MAN13 (graded as BL3Dc, Fig. 1) and it also enabled the derivation of the two sibling lines MAN11 and 12 from a single IVF treatment cycle (Additional file 1: Table S1, Fig. 1). With the availability of a new version of hESF9 (HES-V2) in which the trace level of bovine serum albumin (BSA) was replaced with recombinant human albumin, the totally xeno-free clinical-grade hESC lines MAN15–16 (Additional file 1: Table S1, Fig. 1) were derived. Importantly, both MAN15 and 16 were derived from clinically discarded embryos after an extended period of in vitro culture in clinic (at day 6). All lines are registered and have now been accepted by the UK Stem Cell Bank [14, 15] and are fully compliant with the EUTCD and requirements of UK regulatory authorities including HFEA and HTA, as described recently by De Sousa et al. [13].

#### Characterisation of pluripotency and differentiation in vitro and in vivo

**Fig. 2:**
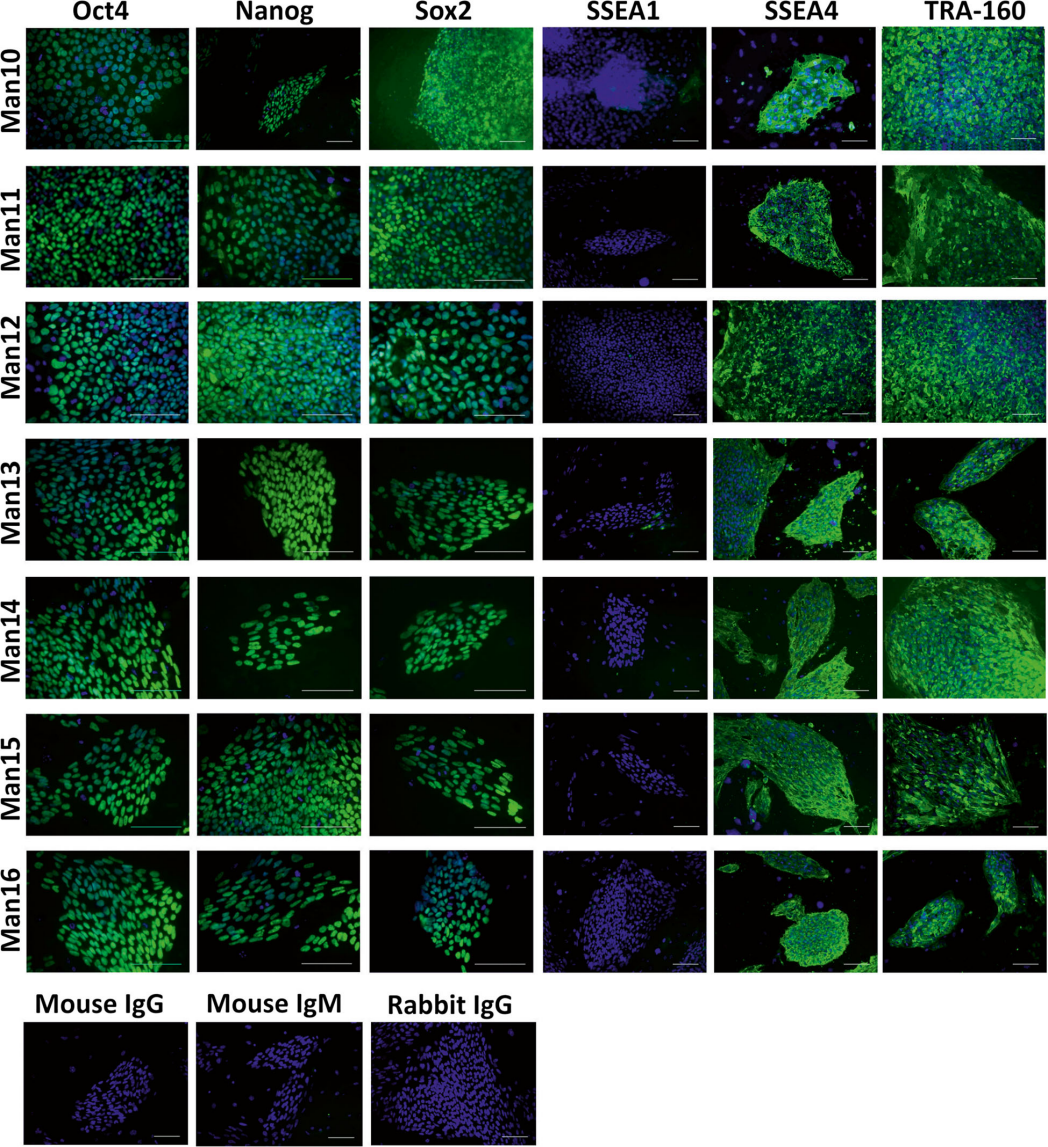
Immunostaining of MAN lines 10–16 for pluripotency markers. At approximately p10 after 5 days of culture on human dermal fibroblasts (hDFs), positive pluripotency markers (*green*) were assessed including OCT4, Nanog, SOX2, SSEA4 and TRA160. The differentiation marker SSEA1 was used as a negative control with DAPI (*blue*) as a counterstain. Isotype controls were mouse IgG, mouse IgM and rabbit IgG. Scale bars represent 100 μM

**Fig. 3:**
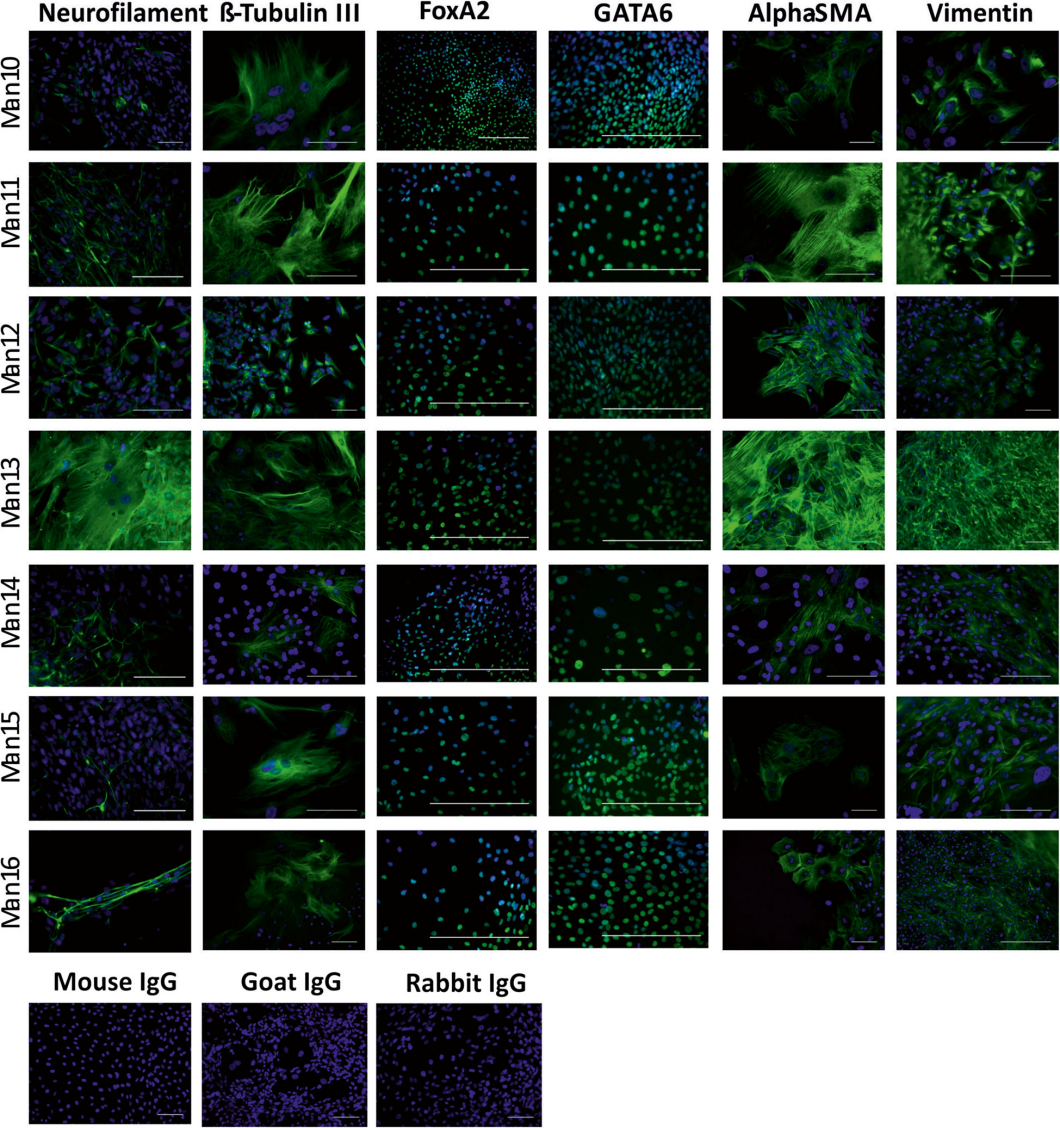
Immunostaining of in vitro differentiated cells from MAN lines 10–16. hESCs were subjected to in vitro differentiation via embryoid body formation and each line analysed by immunocytochemistry for two markers of each germ cell layer (*green*) with DAPI (*blue*) as a counterstain. Ectoderm markers: β-tubulin III and neurofilament; mesoderm markers alpha-smooth muscle actin (alpha-SMA) and vimentin; endoderm markers: GATA6 and FOXA2. Scale bars represent 100uM

**Fig. 4:**
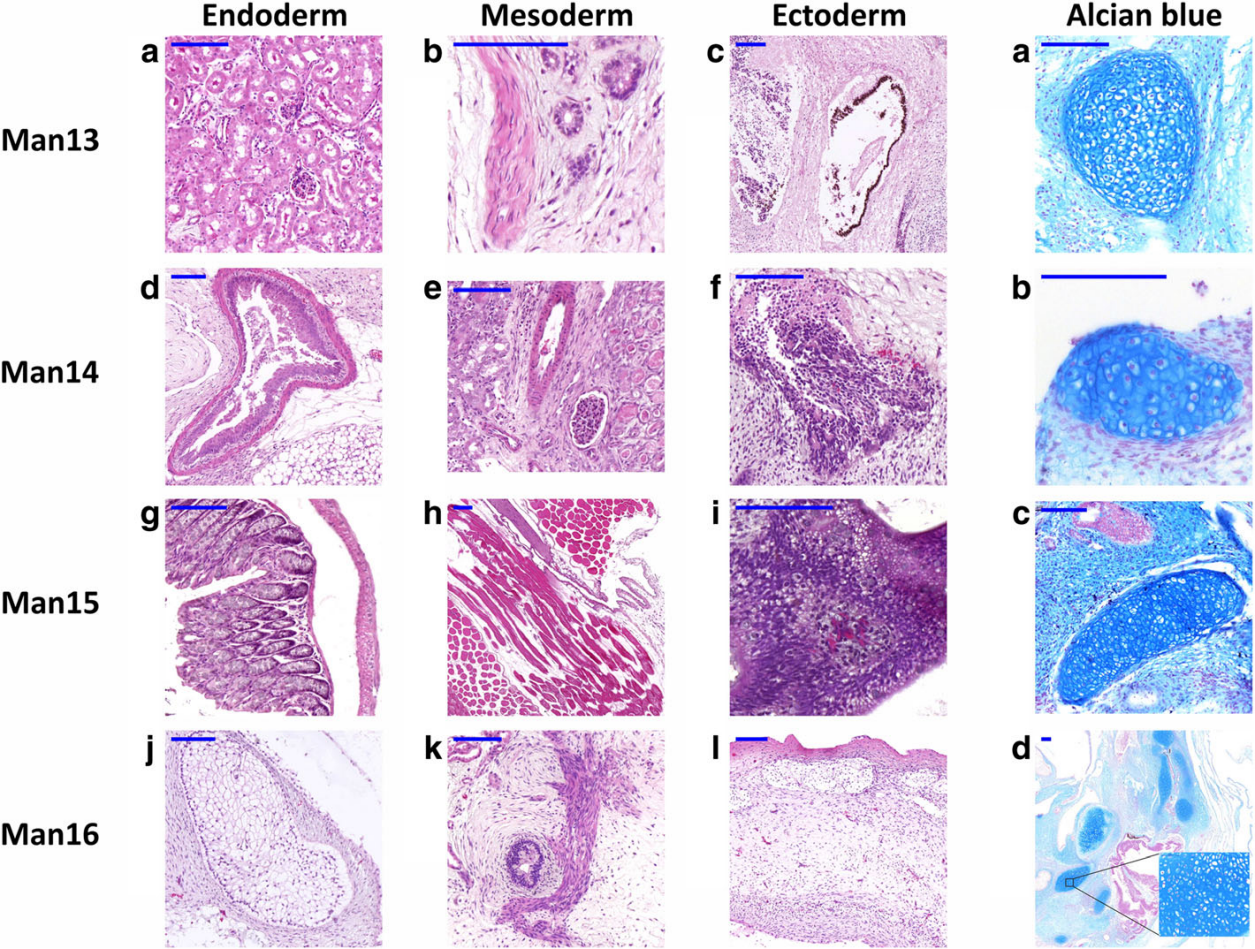
Histological sections of teratomas derived from MAN lines 13–16. The teratomas were formed by subcutaneous injection of MAN13–16 cells under the kidney capsule of a SCID mouse. Stained sections show hematoxylin and eosin staining (A-L) of structures representative of the three germ layers: endoderm (A, D, G, J), mesoderm (B, E, H, K) and ectoderm (C, F, I, L) for MAN13 (A-C), MAN14 (D-F), MAN15 (G-I) and MAN16 (J-L). A, Kidney from endoderm; B, smooth muscle from mesoderm; C, retinal pigment epithelium from ectoderm; D, oesophagus from endoderm; E, artery from mesoderm; F, primitive neuroepithelium from ectoderm; G, intestine from endoderm; H, skeletal muscle from mesoderm; I, neural epithelium from ectoderm; J, sebaceous gland from endoderm; K, smooth muscle from mesoderm; L, skin tissue from ectoderm. a-d Alcian blue-fast red staining showing cartilage (mesoderm) in (a) MAN13, (b) MAN14, (c) MAN15 and (d) MAN16. Scale bars represent 100uM

Standard characterisation for the hESC lines was performed. All showed positive (at least 70% of cells) expression of pluripotent markers including OCT-4, SOX2, NANOG, SSEA-4, TRA-1–60 and lack of expression of SSEA1 (Fig. 2). Following embryoid body (EB)-mediated in vitro differentiation, all lines expressed the following markers of the three germ layers: ectoderm - beta-tubulin III, neurofilament; mesoderm - alpha-smooth muscle actin, vimentin; and endoderm - GATA6, FOXA2 (Fig. 3 and Additional file 1: Table S1). The teratoma assay was performed for MAN13–16 and all gave teratomas with representation of all three germ layers (Fig. 4).

### Genetic testing

**Fig. 5:**
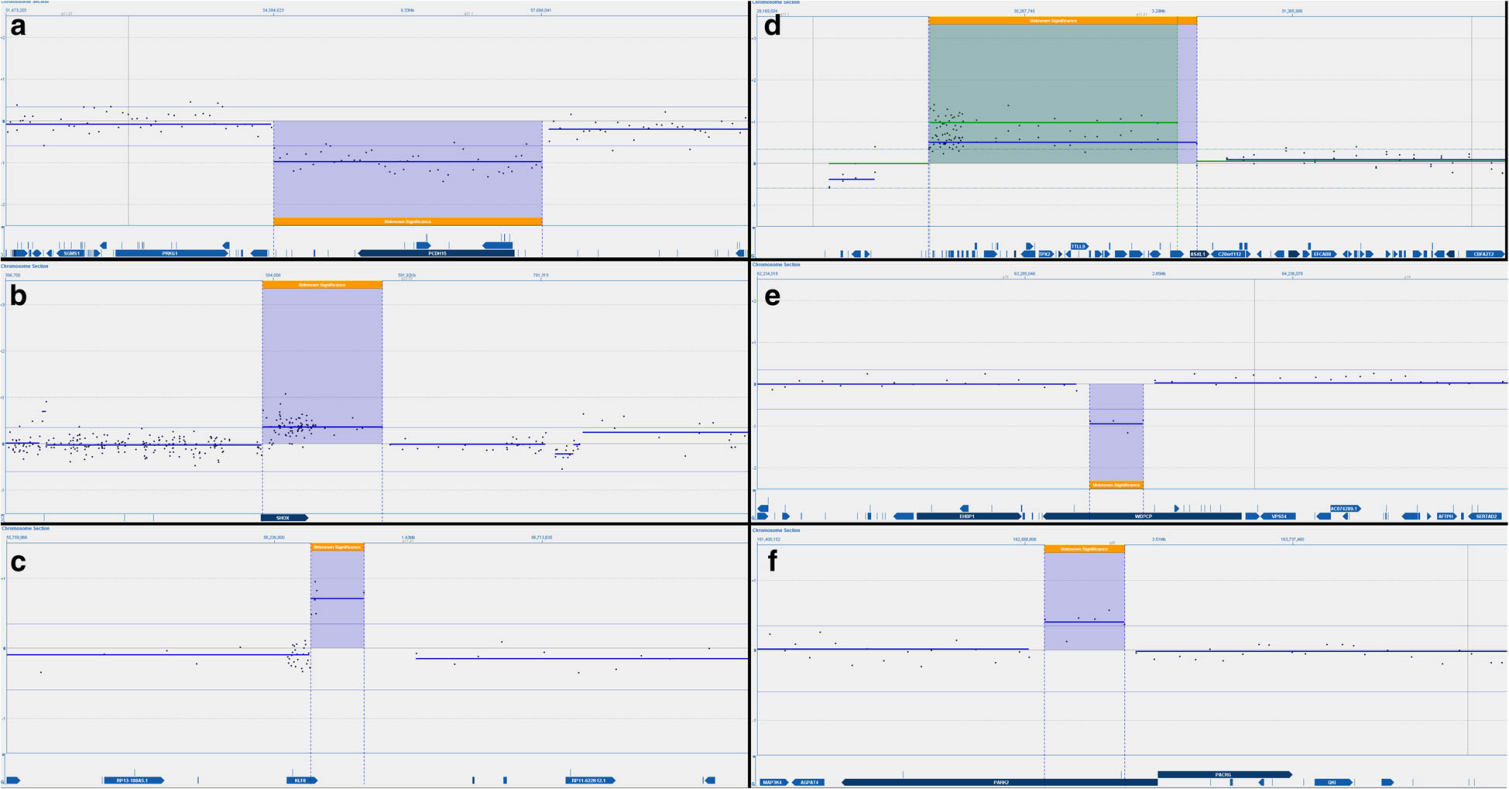
Copy number variants of unknown clinical significance. Data for individual microarray probes are represented by *dots* and plotted on a log_2_ scale of the ratio of hESC DNA/reference DNA. Sub-images are not to scale. Aberrations detected by Cytosure™ Interpret software are visible as *shaded segments* and the average log_2_ ratio of these segments is indicated by *thick solid lines*. **a** Approximately 3.1–3.2 Mb loss of 10q21.1 seen in MAN10 (**b**) approximately 87–94 kb gain of Xp22.33 seen in MAN11 (**c**) approximately 94–189 kb gain of Xp11.21 seen in MAN13 (**d**) gain of 20q11.21 seen in MAN13 (*blue data* approximately 1.1–1.4 Mb ) and MAN14 (*green data* approximately 1.01–1.1 Mb) (**e**) approximately 187–277 kb loss of 2p15 seen in MAN15 (**f**) approximately 346–465 kb gain of 6q26 seen in MAN15

All lines showed normal karyotypes, except MAN11 in which some cells carried a translocation between chromosomes 5q and 9q, with MAN11 and 14 being female and the other lines male (Table 1, Additional file 1: Figure S1). We also performed high-resolution CGH array screening on all lines. In MAN13 and MAN14, CGH arrays identified a gain of approximately 1 Mb of chromosome 20q11.21 common among hESCs. In all other hESC cultures tested, no plausible pathogenic variants were identified based on current evidence. In addition to the detection of benign copy number variations (CNVs) which are present in control populations at a frequency greater than 1% [31], the clinical significance of some variants in MAN10, MAN11, MAN13 and MAN15 were classified as unknown (Table 1, Fig. 5) because their frequency in control populations is less than 1% and/or no evidence is available which suggests they have health implications at the zygosity indicated by the microarray data.

**Table 1:** Genetic screening by karyotype and CGH array

Line	Karyotype	CGH summary	Variants of unknown clinical significance	20q11.21-acquired culture adaptation
MAN10 p14^*^	Normal 46 XY	Male profile	arr[hg19] 10q21.1(54,584,623-57,696,041)x1	
MAN11 p14	46,XX,t(5;9) [13]/46,XX [7]	Female profile:	arr[hg19] Xp22.33(586,009-673,701)x3	
MAN12 p14	Normal 46 XY	Male profile:	None detected	
MAN13 p24	Normal 46 XY	Male profile:	arr[hg19] Xp11.21(56,301,224-56,395,414)x2	arr[hg19] 20q11.21(29,873,327-30,971,568)x3
MAN14 p20	Normal 46 XX	Female profile:	None detected	arr[hg19] 20q11.21(29,877,879-30,889,947)x4
MAN15 p16	Normal 46 XY	Male profile	arr[hg19] 2p15(63,514,505-63,702,363)x1, 6q26(162,654,003-163,000,271)x3	
MAN16 p16	Normal 46 XY	Male profile:	None detected	

^*^Passage number at which cells were analysed

*CGH* comparative genomic hybridisation

### HLA typing

HLA typing was performed on all lines (Table 2). HLA types obtained were compared with published data [32] to assess the proportion of the local population for whom there would be a degree of histocompatibility. The UK population data is derived from individuals of varying native and mixed heritage representative of the national population. Assessment of the HLA specificities across MAN10–16 suggested that a combination of these lines would cover 66.8% of this population for HLA-A, 39.9% for HLA-B, 55.0% for HLA-C, 61.2% for HLA-DRB1 and 95% for HLA-DQB1 (Table 2). A calculated reaction frequency tool [33] can be used to assess the percentage population frequency of HLA antigens present in each cell line within the UK population. Although this tool is more commonly used to assess HLA-specific antibody profiles for reactivity against potential donor organs, it can also be used as an assessment of the potential presence of antigens expressed by these cell lines within the UK population.

**Table 2:** HLA typing

Cell ID	HLA-A*	HLA-B*	HLA-C*	HLA-DRB1*	HLA-DQB1*	cRF% HLA class I	cRF% HLA class I + II
MAN10	02, 68	14, 44	05, 08	04:01, 13:03	03:01	71	86
MAN11	02, 24	35, 44	04, 05	04:01, 11:04	03:01	77	87
MAN12	01, 02	37, 44	05, 06	04:01	03:01	85	90
MAN13	01, 03	07, 35	04, 07	03:01, 04:04	02:01, 03:02	80	87
MAN14	02	07, 35	07, 16	01:01, 03:02	04:02, 05:01	89	93
MAN15	01, 24	07	07	15:01	06:02	86	96
MAN16	01, 02	08, 49	07	03:01, 11:01	02:01, 03:01	71	81

*HLA types of cell lines MAN10–16 obtained using LABType®SSO (One Lambda Inc, Waltham, MA, USA). cRF% is the calculated reaction frequency and represents the cumulative frequency of the HLA antigens present on each single cell line in the UK population

*HLA* human leucocyte antigen

#### Haplotype analysis

A feature of the HLA system is tight linkage disequilibrium across the major histocompatibility complex where the HLA genes are encoded. This creates linked inheritance of HLA specificities which are passed “en bloc” to progeny as maternal and paternal haplotypes. Immunological studies involving shared haplotypes are valuable because of the shared inheritance of all other genes encoded on the same inherited strand of the MHC, many of which encode products involved in the immune response. Although inherited haplotypes could not be confirmed in the sibling cell lines MAN11 and MAN12 due to the absence of parental material to validate the proposed inheritance patterns, using the local population data available, it was possible to infer haplotypes present in this sibling pair. MAN11 and MAN12 appeared to have three distinct inherited haplotypes:

- HLA-A*02, B*44, C*05, DRB1*04:01, DQB1*03:01
- HLA-A*24, B*35, C*04, DRB1*11:04, DQB1*03:01
- HLA-A*01, B*37, C*06, DRB1*04:01, DQB1*03:01

MAN11 has inherited haplotypes (a) and (b); while MAN12 has inherited haplotypes (a) and (c). Haplotype (a) is a relatively common HLA haplotype, with a population frequency of 3.9% in northwest England. It is likely, therefore, that MAN10 may also have independently inherited this haplotype from a separate ancestral source, although this cannot be confirmed. Other common haplotypes include the HLA-A*01, B*08, C*07, DRB1*03, DQB1*02 haplotype (approximately 15%), which occurs at increased frequency in several autoimmune disorders and is commonly referred to as “the autoimmune haplotype”. It is likely that MAN15 carries this haplotype.

## Discussion

The clinical-grade hESC lines described here, are of the highest quality yet produced for clinical therapeutic use, for the following reasons: (i) they have been derived from sperm and eggs procured and embryos generated in an IVF laboratory specifically designed and operated to GMP standards, as well as derivation and expansion under a GMP Quality Management System, and (ii) they have been sourced from fresh embryos deemed unsuitable for clinical treatment, and therefore have the highest possible ethical and moral status in terms of embryo viability since these embryos were not intended to establish a pregnancy. This status lowers a significant hurdle to the widespread use of hESCs to provide allogeneic tissue banks for regenerative medicine applications, balanced against the opposition to the use of embryo-derived stem cell lines in many parts of the world. Although autologous stem cell therapies using induced pluripotent stem cells (hiPSCs) and/or somatic cell nuclear transfer-derived hESCs are possible avenues for regenerative medicine therapies, a likely route for widespread, cost-effective application of many hPSC-derived cell replacement therapies is via allogeneic tissue banks of high-quality, carefully screened, HLA-matched hESC lines, as reflected by the latest progress in the world’s first clinical trial with hiPSCs (derived retinal pigmented epithelial cells, RPE; [29]). It is important to note that the world’s first clinical trial with autologous hiPSCs-RPE has been temporarily halted currently due to mutations found in the hiPSCs and product cells, but not the corresponding patient fibroblasts, as well as regulatory changes and other practical considerations including the cost, and that it is likely the trial will be switched to the use of allogeneic hiPSCs in future [34, 35]. A large number of clinical trials have been initiated recently using hESC-derived progenitor cells for conditions such as spinal cord injury, macular degeneration, diabetes, and heart failure, demonstrating the considerable existing and potential demand [3, 36–39]. However to date, fewer than 50 clinical-grade hESC lines exist around the world, thus there is an urgent need for robust protocols to derive additional hESC lines at the highest possible cellular, genetic and ethical standards. In the present study, we have successfully generated seven high-quality hESC lines of complete GMP status from embryo procurement, among which two lines are totally xeno-free (MAN15 and 16). In addition, MAN15 is homozygous at all HLA loci except HLA-A.

During infertility treatment in the UK, one or two embryos are routinely selected as the ‘best’ ones to transfer in each IVF cycle. Any embryos that are not transferred immediately may be cryopreserved for later use. Embryos that score low on developmental and morphological criteria [40] are classed as unsuitable for clinical use and are ‘allowed to perish’ and discarded immediately. Infrastructure funding was provided in the UK to enable IVF laboratories to be built and operated at GMP to facilitate the ‘IVF-stem cell interface’, increase the supply of fresh ‘spare’ embryos and, crucially, to ensure that the source material for derivation of clinical-grade hESC lines was at GMP standard from the procurement of gametes (eggs and sperm) onwards. Cryopreserved embryos tend to become available many years later, as in the UK patients can store embryos for up to 10 years. Indeed, most embryos used for hESC research, especially for clinical-grade hESC derivations, came from long-term cryopreservation storage [16–18, 41]. In this situation, there are some complicated clinical, technical, social and ethical factors associated with both the donation of embryos and of the derivation of hESCs, given that there are rarely authentically ‘surplus’ embryos from infertility treatments [25, 27]. This controversy over the source of hESCs may not be avoided completely even by the way of cleavage-stage embryo single-cell biopsy, since it is difficult to determine the damage incurred by the remaining embryo following the removal of cells for hESC derivation [42, 43]. Thus we argue that it is more acceptable to use fresh and poor-quality embryos which are routinely discarded, than to use or damage good-quality embryos.

For the purposes of deriving clinical-grade hESC lines, which might be used in the treatment of thousands of patients, using embryos generated at GMP standards that are higher than those required for routine IVF treatment provides an important additional aspect of quality. The human embryo undergoes a complete reorganisation of the genome and epigenome immediately following fertilisation and before the point of hESC derivation, and there is evidence that these processes are disrupted by environmental variables in the IVF laboratory (reviewed in [44]). Therefore the control of these variables (e.g. airborne particulates and biological organisms, incubator temperatures, media composition and pH) to GMP standards becomes very significant in consideration of hESC quality, arguably as important as GMP standards during subsequent hESC derivation and cell line maintenance.

Although studies have shown that even poor-quality or arrested embryos can give rise to hESC lines [45–47], the success rate is usually very low. Deriving clinical-grade lines is lower still than that of research grade, even with good-quality frozen embryos, as the translation of derivation systems to xeno-free culture conditions is associated with reduced success [17]. Therefore, it requires the development of a novel and robust derivation culture system to maximise use of the pluripotent stem cell population in fresh discarded and poor-quality embryos in the derivation of clinical-grade hESC lines. In the present study, we have established a unique and highly efficient system using sequential commercial and chemically defined media hESF9 and TeSR2 on GMP-grade hDFs as feeder cells under a stringent GMP Quality Management System and xeno-free eventually.

Ideally, clinical-grade hESC lines would be derived in a completely defined culture system without feeder cells. A single study by Ludwig et al. [48] showed the derivation of two hESC lines in original TeSR2 medium with a complex of recombinant cell extracellular matrix components. Very recently, Rodin et al. [49] have developed a robust chemically defined culture system with a matrix mixture of laminin (LN-521) and E-cadherin that allows highly stable hESC cultures and derivation of new hESC lines from isolated ICMs or even from a single blastomere biopsied from an eight-cell in vitro fertilisation (IVF) embryo. However, those derivations were not performed within a GMP-cleanroom laboratory. Although it appears possible to establish new research-grade hESC lines in such a feeder-free system, a fully GMP-compliant derivation system has yet to be developed [7, 17, 50]. Moreover, good-quality frozen embryos were used to isolate the well-developed ICMs, aided sometimes with a specialist laser-aided micromanipulation system [49, 50]. We have tried several feeder-free media and different extracellular matrix components without feeder cells and found none of the feeder-free systems supported the derivation of hESC lines from the fresh discarded embryos we have collected (data not shown). We speculated that it might be possible to derive new hESC lines from such fresh discarded embryos in xeno-free medium but on human feeder cells, providing a more complex environment to initiate the growth of ICMs from the combination of medium and feeder cells together. We used GMP-grade hDFs, as human skin fibroblasts have also been used by other groups. However, our results showed that hESC derivation was not possible with any single medium we tested on the hDFs. Hakala et al. [51] have compared nine media for maintenance culture of existing hESC lines and found that none of the xeno-free media they tested including TeSR2 is supportive with human skin fibroblasts. However, our results have shown that the sequential use of media HES-V2 (and its predecessor hESF9) and TeSR2 can successfully support the establishment of hESC lines. Under our conditions, both hDF lines supported successful derivation of hESCs: MAN9–15 were derived on one hDF line while MAN16 was derived on the other, and the new derived lines can be maintained on both hDF feeder lines (e.g. MAN13). Others have also found it beneficial to use sequential medium. For instance, initial ICM growth in Quinn’s Advantage Protein Plus Blastocyst Medium followed by TeSR2 has previously been used for clinical-grade hESC derivation [8, 18]. This likely reflects the different conditions required for ICM survival and adaptation to ex vivo culture and expansion of stem cells. Indeed our derivation system with the sequential use of commercially available media, HES-V2 and TeSR2, is robust, as in our hands even the ICMs with few or no distinguishable cells (MAN13, 15 and 16) from discarded surplus embryos could be rescued and expanded successfully to establish cell lines. It is extremely encouraging that fresh clinical discarded embryos, though too poor in quality for fertility treatment, can still give rise to high-quality clinical-grade hESC lines. The successful derivations must have depended on very few remaining/survived ICM cells. In fact, it has been shown that even a single ICM cell is able to proliferate and form a colony of ESCs under suitable conditions in the mouse and human [52, 53].

It is imperative that hESCs used for clinical therapy are of the highest possible genetic quality. Advances in technology and an increasing cytogenetic knowledge base demand high-quality analysis, hence we have carried out high-resolution array CGH on all lines. Reassuringly, no currently plausible pathogenic variants were identified in any of our hESC lines. Minor aberrations were observed in MAN10, MAN11, MAN13 and MAN15 but without any associated evidence of pathogenicity. Moreover, the microarray data did not indicate the presence of mosaic aberrations, suggesting (within the limitations of microarray technology) that these sequence variants were likely to have been present in the donated embryo and were not induced during derivation of hESCs. Indeed variants shown in Fig. 5a, b, c and f have all been observed as germline variants previously at a low frequency in the general population [31], or in populations in whom microarray testing has been undertaken [54]. The exception to this is the gain of chromosome 20 seen in MAN13 and MAN14; this is a feature of culture adaptation commonly seen in hESC lines [55] and unlikely to be of clinical concern. In addition to array CGH, all lines show normal karyotype bar the balanced translocation seen in some cells of MAN11. However as the CGH has shown no imbalance at the breakpoints, this is also unlikely to be clinically significant. MAN11 and MAN12 have also been recently screened by whole genome single nucleotide polymorphism (SNP) array analysis, showing no copy number variation (CNV) in MAN12, and only a minor CNV in MAN11 which is present in normal healthy individuals [12].

Optimally, transplantation of embryonic stem-derived cells would require availability of histocompatible hESC lines for every patient. Since significant differences in histocompatibility types exist among various ethnic populations, establishment of a bank of lines representing human genetic diversity will be vital for successful therapeutic purposes. Using a combination of our lines MAN10–16, HLA specificities present in 66.8% of the UK population for HLA-A, 39.9% for HLA-B, 55.0% for HLA-C, 61.2% for HLA-DRB1 and 95% for HLA-DQB1 would be covered. Restricted presentation of HLA class II antigens (including HLA-DR, HLA-DQ) would suggest that matching for HLA class I specificities would normally take priority for the use of hESCs. It follows that for maximum histocompatibility, it is useful to have cells which are homozygous for HLA, inheriting two copies of the same haplotype, so that a single specificity is present at each locus, reducing the likelihood of direct allorecognition and rejection. These cells are extremely rare, originating most commonly in consanguineous families, but also arising by chance. MAN15 is homozygous at all loci except HLA-A, and therefore has a restricted capacity to provoke an immune response compared with heterozygous cell lines. This feature is advantageous for use in studies of antigen presentation, where reactivity can be assessed without ambiguity, and in cellular therapies, as the presence of only one specificity at each locus minimises the risk of immunological rejection. Thus, the newly derived MAN10–16 lines, especially MAN15, make a significant contribution to the effort to provide an allogenic tissue bank with wide coverage.

## Conclusions

To our knowledge, the present study, for the first time, reports the successful derivation of high-quality clinical-grade hESC lines from fresh discarded and poor-quality embryos that were originally generated in a GMP-grade IVF laboratory. As hESCs are natural pluripotent stem cells, they remain the gold standard in this field. hESC-derived cells have been used in clinical trials and the preliminary results are promising [3, 21]. Our culture system has allowed us to derive the highest standard of clinical-grade hESC lines at a more widely acceptable ethical standard than any yet reported. This protocol coupled with our existing bank of well-characterised hESC lines now provides a robust platform to go on to derive the larger number of exemplary-quality clinical-grade lines required for tissue-banking purposes, from GMP gametes and embryos procured in the most ethically acceptable manner. As such this represents an important step towards widespread use of hESCs in allogeneic cell replacement therapy.

## Additional file

Additional file 1: Supplemental information includes supplemental experimental procedures, one figure and one table. (ZIP 292 kb)

## Abbreviations

CGH: Comparative genomic hybridisation
CNV: Copy number variation
EUTCD: European Union Tissues and Cells Directive
GMP: Good Manufacturing Practice
hDF: Human dermal fibroblast
hESC: Human embryonic stem cell
HFEA: Human Fertilisation and Embryology Authority
HLA: Human leucocyte antigen
hPSF: Human placental stromal fibroblasts
HTA: Human Tissue Authority
ICM: Inner cell mass
IVF: In vitro fertilisation
MEF: Mouse embryonic fibroblasts
MHRA: Medicines and Healthcare Regulatory Agency
UK: United Kingdom

## References

[1] Evans MJ, Kaufman MH (1981). Establishment in culture of pluripotential cells from mouse embryos. Nature.

[2] Thomson JA, Itskovitz-Eldor J, Shapiro SS, Waknitz MA, Swiergiel JJ, Marshall VS, Jones JM (1998). Embryonic stem cell lines derived from human blastocysts. Science.

[3] Neofytou E, O’Brien CG, Couture LA, Wu JC (2015). Hurdles to clinical translation of human induced pluripotent stem cells. J Clin Investig.

[4] Unger C, Skottman H, Blomberg P, Dilber MS, Hovatta O (2008). Good manufacturing practice and clinical-grade human embryonic stem cell lines. Hum Mol Genet.

[5] Arabadjiev B, Petkova R, Chakarov S, Momchilova A, Pankov R (2010). Do we need more human embryonic stem cell lines?. Biotechnol Biotechnol Equip.

[6] Fraga AM, de Araujo ES S, Stabellini R, Vergani N, Pereira LV (2011). A survey of parameters involved in the establishment of new lines of human embryonic stem cells. Stem Cell Rev Rep.

[7] Damdimopoulou P, Rodin S, Stenfelt S, Antonsson L, Tryggvason K, Hovatta O (2016). Human embryonic stem cells. Best Pract Res Clin Obstetr Gynaecol.

[8] Ilic D, Stephenson E, Wood V, Jacquet L, Stevenson D, Petrova A, Kadeva N, Codognotto S, Patel H, Semple M (2012). Derivation and feeder-free propagation of human embryonic stem cells under xeno-free conditions. Cytotherapy.

[9] Fraga AM, de Araujo ESS, Stabellini R, Vergani N, Pereira LV (2012). Establishment of new lines of human embryonic stem cells: evolution of the methodology. Methods Mol Biol.

[10] Vicenta Camarasa M, Miguel Galvez V, Brison DR, Bachiller D (2012). Optimized protocol for derivation of human embryonic stem cell lines. Stem Cell Rev Rep.

[11] Desai N, Rambhia P, Gishto A (2015). Human embryonic stem cell cultivation: historical perspective and evolution of xeno-free culture systems. Reprod Biol Endocrinol.

[12] Canham MA, Van Deusen A, Brison DR, De Sousa PA, Downie J, Devito L, Hewitt ZA, Ilic D, Kimber SJ, Moore HD (2015). The molecular karyotype of 25 clinical-grade human embryonic stem cell lines. Sci Rep.

[13] De Sousa PA, Downie JM, Tye BJ, Bruce K, Dand P, Dhanjal S, Serhal P, Harper J, Turner M, Bateman M (2016). Development and production of good manufacturing practice grade human embryonic stem cell lines as source material for clinical application. Stem Cell Res.

[14] UK Stem Cell Bank - Our research - Medical Research Council [https://www.mrc.ac.uk/research/facilities-and-resources-for-researchers/stem-cell-bank/] Accessed 4 Apr 2017.

[15] Home · hPSCreg [https://hpscreg.eu/]. Accessed 4 Apr 2017.

[16] Crook JM, Peura TT, Kravets L, Bosman AG, Buzzard JJ, Horne R, Hentze H, Dunn NR, Zweigerdt R, Chua F (2007). The generation of six clinical-grade human embryonic stem cell lines. Cell Stem Cell.

[17] Tannenbaum SE, Turetsky TT, Singer O, Aizenman E, Kirshberg S, Ilouz N, Gil Y, Berman-Zaken Y, Perlman TS, Geva N (2012). Derivation of xeno-free and GMP-grade human embryonic stem cells - platforms for future clinical applications. PLoS ONE.

[18] Stephenson E, Jacquet L, Miere C, Wood V, Kadeva N, Cornwell G, Codognotto S, Dajani Y, Braude P, Ilic D (2012). Derivation and propagation of human embryonic stem cell lines from frozen embryos in an animal product-free environment. Nat Protoc.

[19] De Sousa PA, Tye BJ, Bruce K, Dand P, Russell G, Collins DM, Greenshields A, McDonald K, Bradburn H, Canham MA (2016). Derivation of the clinical grade human embryonic stem cell line RCe013-A (RC-9). Stem Cell Res.

[20] De Sousa PA, Tye BJ, Bruce K, Dand P, Russell G, Collins DM, Greenshields A, McDonald K, Bradburn H, Allan D (2016). Derivation of the clinical grade human embryonic stem cell line RCe021-A (RC-17). Stem Cell Res.

[21] Trounson A, DeWitt ND (2016). Pluripotent stem cells progressing to the clinic. Nat Rev Mol Cell Biol.

[22] Osafune K, Caron L, Borowiak M, Martinez RJ, Fitz-Gerald CS, Sato Y, Cowan CA, Chien KR, Melton DA (2008). Marked differences in differentiation propensity among human embryonic stem cell lines. Nat Biotechnol.

[23] Oldershaw RA, Baxter MA, Lowe ET, Bates N, Grady LM, Soncin F, Brison DR, Hardingham TE, Kimber SJ (2010). Directed differentiation of human embryonic stem cells toward chondrocytes. Nat Biotechnol.

[24] Taylor CJ, Bolton EM, Pocock S, Sharples LD, Pedersen RA, Bradley JA (2005). Banking on human embryonic stem cells: estimating the number of donor cell lines needed for HLA matching. Lancet.

[25] De Sousa PA, Gardner J, Sneddon S, Pells S, Tye BJ, Dand P, Collins DM, Stewart K, Shaw L, Przyborski S (2009). Clinically failed eggs as a source of normal human embryo stem cells. Stem Cell Res.

[26] Camarasa MV, Kerr RW, Sneddon SF, Bates N, Shaw L, Oldershaw RA, Small F, Baxter MA, McKay TR, Brison DR, Kimber SJ (2010). Derivation of Man-1 and Man-2 research grade human embryonic stem cell lines. In Vitro Cell Dev Biol Anim.

[27] Brison DR, Lieberman BA (2003). Use eggs, not embryos, to derive stem cells. Br Med J.

[28] Candela Crocco M, Fratnz N, Bos-Mikich A (2013). Substrates and supplements for hESCs: a critical review. J Assist Reprod Genet.

[29] McKay TR, Camarasa MV, Iskender B, Ye J, Bates N, Miller D, Fitzsimmons JC, Foxler D, Mee M, Sharp TV (2011). Human feeder cell line for derivation and culture of hESc/hiPSc. Stem Cell Res.

[30] Baxter MA, Camarasa MV, Bates N, Small F, Murray P, Edgar D, Kimber SJ (2009). Analysis of the distinct functions of growth factors and tissue culture substrates necessary for the long-term self-renewal of human embryonic stem cell lines. Stem Cell Res.

[31] Database of genomic variants [http://dgv.tcag.ca/dgv/app/home] Accessed 4 Apr 2017.

[32] The allele frequency net database - allele, haplotype and genotype frequencies in worldwide populations [http://allelefrequencies.net/] Accessed 4 Apr 2017.

[33] NHS Blood and Transplant - Organ Donation and Transplantation Clinical Site - H&I Information [http://www.odt.nhs.uk/transplantation/histocompatibility-and-immunogenetics/handi-information/] Accessed 4 Apr 2017.

[34] Garber K (2015). RIKEN suspends first clinical trial involving induced pluripotent stem cells. Nat Biotechnol.

[35] Scudellari M (2016). How iPS cells changed the world. Nature.

[36] Menasche P, Vanneaux V, Hagege A, Bel A, Cholley B, Cacciapuoti I, Parouchev A, Benhamouda N, Tachdjian G, Tosca L (2015). Human embryonic stem cell-derived cardiac progenitors for severe heart failure treatment: first clinical case report. Eur Heart J.

[37] Song WK, Park K-M, Kim H-J, Lee JH, Choi J, Chong SY, Shim SH, Del Priore LV, Lanza R (2015). Treatment of macular degeneration using embryonic stem cell-derived retinal pigment epithelium: preliminary results in Asian patients. Stem Cell Rep.

[38] Schwartz SD, Regillo CD, Lam BL, Eliott D, Rosenfeld PJ, Gregori NZ, Hubschman J-P, Davis JL, Heilwell G, Spirn M (2015). Human embryonic stem cell-derived retinal pigment epithelium in patients with age-related macular degeneration and Stargardt’s macular dystrophy: follow-up of two open-label phase 1/2 studies. Lancet.

[39] Schulz TC (2015). Concise review: manufacturing of pancreatic endoderm cells for clinical trials in type 1 diabetes. Stem Cells Transl Med.

[40] Balaban B, Brison D, Calderon G, Catt J, Conaghan J, Cowan L, Ebner T, Gardner D, Hardarson T, Lundin K (2011). Istanbul consensus workshop on embryo assessment: proceedings of an expert meeting. Reprod Biomed Online.

[41] Ehrich K, Williams C, Farsides B (2010). Fresh or frozen? Classifying ‘spare’ embryos for donation to human embryonic stem cell research. Soc Sci Med.

[42] Green RM (2007). Can we develop ethically universal embryonic stem-cell lines?. Nat Rev Genet.

[43] Hyun I (2010). The bioethics of stem cell research and therapy. J Clin Investig.

[44] Brison DR, Roberts SA, Kimber SJ (2013). How should we assess the safety of IVF technologies?. Reprod Biomed Online.

[45] Gavrilov S, Marolt D, Douglas NC, Prosser RW, Khalid I, Sauer MV, Landry DW, Vunjak-Novakovic G, Papaioannou VE (2011). Derivation of two new human embryonic stem cell lines from nonviable human embryos. Stem Cells Int.

[46] O’Leary T, Heindryckx B, Lierman S, Van der Jeught M, Menten B, Deforce D, Cornelissen R, Lopes SCS, De Sutter P (2011). The influence of early embryo traits on human embryonic stem cell derivation efficiency. Stem Cells Dev.

[47] Zhang X, Stojkovic P, Przyborski S, Cooke M, Armstrong L, Lako M, Stojkovica M (2006). Derivation of human embryonic stem cells from developing and arrested embryos. Stem Cells.

[48] Ludwig TE, Levenstein ME, Jones JM, Berggren WT, Mitchen ER, Frane JL, Crandall LJ, Daigh CA, Conard KR, Piekarczyk MS (2006). Derivation of human embryonic stem cells in defined conditions. Nat Biotechnol.

[49] Rodin S, Antonsson L, Niaudet C, Simonson OE, Salmela E, Hansson EM, Domogatskaya A, Xiao Z, Damdimopoulou P, Sheikhi M (2014). Clonal culturing of human embryonic stem cells on laminin-521/E-cadherin matrix in defined and xeno-free environment. Nat Commun.

[50] Rodin S, Antonsson L, Hovatta O, Tryggvason K (2014). Monolayer culturing and cloning of human pluripotent stem cells on laminin-521-based matrices under xeno-free and chemically defined conditions. Nat Protoc.

[51] Hakala H, Rajala K, Ojala M, Panula S, Areva S, Kellomaki M, Suuronen R, Skottman H (2009). Comparison of biomaterials and extracellular matrices as a culture platform for multiple, independently derived human embryonic stem cell lines. Tissue Eng A.

[52] Boroviak T, Loos R, Bertone P, Smith A, Nichols J (2014). The ability of inner-cell-mass cells to self-renew as embryonic stem cells is acquired following epiblast specification. Nat Cell Biol.

[53] Guo G, von Meyenn F, Santos F, Chen Y, Reik W, Bertone P, Smith A, Nichols J (2016). Naive pluripotent stem cells derived directly from isolated cells of the human inner cell mass. Stem Cell Rep.

[54] ClinGen - ClinGen | Clinical Genome Resource [https://www.clinicalgenome.org/] Accessed 4 Apr 2017.

[55] Amps K, Andrews PW, Anyfantis G, Armstrong L, Avery S, Baharvand H, Baker J, Baker D, Munoz MB, Beil S (2011). Screening ethnically diverse human embryonic stem cells identifies a chromosome 20 minimal amplicon conferring growth advantage. Nat Biotechnol.

