# Supplementary material for: High quality clinical grade human embryonic stem cell lines derived from fresh discarded embryos
Source: Stem Cell Res Ther. 2017 Jun 5;8:128. doi: 10.1186/s13287-017-0561-y (PMC5460457; doi:10.1186/s13287-017-0561-y)
Supplement: Additional file 1: — Supplemental information includes supplemental experimental procedures, one figure and one table. (ZIP 292 kb) [file 13287_2017_561_MOESM1_ESM.zip › J Ye et al manuscript Supplemental .docx]

**Additional file**

**High quality clinical grade human embryonic stem cell lines derived from fresh discarded embryos**

**Jinpei Ye^1, 6,*^, Nicola Bates^1, 6,*^, Despina Soteriou^1, 6^, Lisa Grady^1, 6^, Clare Edmond^1, 6^, Alex Ross^2, 6^, Alan Kerby^1^, Philip A. Lewis^1^, Tope Adeniyi^2^, Ronnie Wright^3^, Kay V. Poulton^4^, Marcus Lowe^4^, Susan J. Kimber^1,6^, Daniel R. Brison^2,5,6^**

SUPPLEMENTAL DATA

**Figure S1 Example of normal karyotypes of MAN 14 and 16.**

Normal (a) female and (b) male karyotypes for MAN 14 and 16 are shown respectively. Images were obtained using Metasystems image capture and karyotyping software and are shown at approximately x630 magnification.

**Table S1 Derivation and characterisation record of clinical grade hESC lines**

| **hESC line** | **Stage of donated embryo** | **HDF line** | **GMP** | **Totally Xeno-free** | **Pluripotency markers by immunochemistry** | | | | | | **EB in vitro Mesoderm** | | **EB in vitro Ectoderm** | | **EB in vitro Endoderm** | | **Teratoma** | **Karo-typing** | **CGH** | **HLA** | **Mycoplasma** |
| --- | --- | --- | --- | --- | --- | --- | --- | --- | --- | --- | --- | --- | --- | --- | --- | --- | --- | --- | --- | --- | --- |
|  |  |  |  |  | **Oct 4** | **Nanog** | **Sox2** | **SSEA1** | **SSEA4** | **Tra160** | **Vimentin** | **Alpha SMA** | **Beta Tublin III** | **Neuro-filiment** | **GATA 6** | **FoxA2** |  |  |  |  |  |
| **MAN 10** | **D3 cleavage** | p106090049 | Y | N | √ | √ | √ | √ | √ | √ | √ | √ | √ | √ | √ | √ |  | √ | √ | √ | Negative |
| **MAN 11** | **D3 cleavage** | p106090049 | Y | N | √ | √ | √ | √ | √ | √ | √ | √ | √ | √ | √ | √ |  | √ | √ | √ | Negative |
| **MAN 12** | **D3 cleavage** | p106090049 | Y | N | √ | √ | √ | √ | √ | √ | √ | √ | √ | √ | √ | √ |  | √ | √ | √ | Negative |
| **MAN 13** | **D4 cleavage** | p106090049 | Y | N | √ | √ | √ | √ | √ | √ | √ | √ | √ | √ | √ | √ | √ | √ | √ | √ | Negative |
| **MAN 14** | **D5 blastocyst** | p106090049 | Y | N | √ | √ | √ | √ | √ | √ | √ | √ | √ | √ | √ | √ | √ | √ | √ | √ | Negative |
| **MAN 15** | **D6 blastocyst** | p106090049 | Y | Y | √ | √ | √ | √ | √ | √ | √ | √ | √ | √ | √ | √ | √ | √ | √ | √ | Negative |
| **MAN 16** | **D6 blastocyst** | p107080110 | Y | Y | √ | √ | √ | √ | √ | √ | √ | √ | √ | √ | √ | √ | √ | √ | √ | √ | Negative |

Note: Y- yes; N- no;
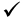
performed/completed.

SUPPLEMENTAL EXPERIMENTAL PROCEDURES

*GMP source of human embryos*

Human embryos discarded from treatment were donated by couples undergoing routine IVF treatment cycles at St Mary’s Hospital, Manchester funded by the UK National Health Service [[1](#_ENREF_1)]. Patient information sheets and consent forms were drawn up according to UK national guidelines as part of the Human Embryonic Stem Cell Co-ordinators (hESCCO) group [[2](#_ENREF_2)] and subsequently the UK National Clinical human Embryonic Stem Cell Forum [[3](#_ENREF_3)]. Informed consent was obtained from all couples who donated their embryos for the present study. In all cases these were the poorest quality embryos remaining from a treatment cycle, after the best embryos had been transferred to the patient or cryopreserved as part of clinical treatment. Embryos were scored for developmental progression and morphological grade using standardised criteria [[4](#_ENREF_4)] and selected for transfer, cryopreservation or “allowed to perish” according to standard operating procedures. Only embryos which were destined by clinical decision to be allowed to perish were selected for stem cell derivation attempts. The clinical decision was made by a member of the clinical embryology team, independent of the stem cell researcher, and patient consenting was performed by a dedicated research nurse independent of both the clinical and research teams. The embryos we used were therefore either collected at Day3/4 (during cleavage) and of poor quality with rarely discernable ICMs after culture to Day 5 or 6, or collected at Day 5/6 (blastocytst stage) and of poor qulaity witout discernable ICMs.

*Embryo culture*

Upon donation to research, embryos were collected in 0.8ml of G1 medium (Vitrolife, Goteborg, Sweden) covered with a thin layer of mineral oil (Ovoil, Origio, Malov, Denmark) in a 5-ml tube previously incubated in 6% CO_2_ and transported to the hESC GMP derivation laboratory in a portable incubator at 37˚C within 1 hour. After examination under stereomicroscopy in a Class II laminar air flow cabinet, the embryos were cultured in drops of 30ul G1 (day 2-3) or G2 (Vitrolife) (day 3 onwards) covered with a thin layer of mineral oil at 37˚C in 6% CO_2_ in a humidified atmosphere until the blastocyst stage. The quality of blastocysts was assessed according to the extent of blastocoel expansion (1: <50%, 2: 50-80%, 3: >80%, 4: hatching/hatched), inner cell mass (A: large compact, B: large loose, C: small/tiny, D: none) and trophoblast (a: extensive and well organised, b: less extensive c: very few cells) morphology as BL (1-4, A-D, a-c), as modified from Stephenson [[5](#_ENREF_5)].

*Culture media*

Various culture media were prepared for the derivation and maintenance of hESC lines at GMP standard. The standard hESC culture medium (HES1) used for derivation and maintenance of hESC lines on mouse embryonic fibroblasts (MEFs) was knockout DMEM (KO-DMEM, Invitrogen, Paisley, UK) supplemented with 20% (v/v) knockout serum replacement (KO-SR, Invitrogen), 10 ng/ml basic fibroblast growth factor (bFGF, Autogen Bioclear), 2 mM L-glutamine, 1% non-essential amino acids (NEAA) and 0.1 mM β-mercaptoethanol and 1% penicillin-streptomycin (Life Tech, Paisley, UK). The xeno-free HES1 (HES1-xf) contained 20% (v/v) xeno-free KO-SR (Invitrogen) instead of KO-SR. An alternative standard hESC culture medium (HES2) contained the basal medium DMEM/F12 (1:1, Invitrogen) instead of KO-DMEM and was used for hESC maintenance only with MEFs. The standard medium for hESC maintenance without feeder cells was Advanced DMEM/F12 (medium AD, Invitrogen) supplemented with 0.1% (w/v) BSA (Sigma), 2mM L-glutamine and 1% NEAA, 0.1mM β-mercaptoethanol, 1x lipid supplement, 1x N2 supplement,1x B27 supplement, 20ng/ml bFGF, 10ng/ml Activin A (R&D) and 2ng/ml NT4 (Preprotech). Other media used for hESC line derivation and culture included commercially ready-made and fully defined medium hESF9 (Cell Science & Technology Institute, Inc., Sendai, Japan) which contained BSA as the only traced xeno-element and its totally xeno-free version medium hESF v2 in which all supplemented compounds were human re-combinant, as well as media TeSR2 (StemCell Technologies), NutriStem (Biological Industries), and StemPro (Invitrogen). The medium for the culture of MEFs (medium MF) was high glucose DMEM supplemented with 10% foetal bovine serum (FBS), 2 mM L-glutamine, and 1% penicillin-streptomycin. The medium for xeno-free culture of hDFs (medium HF) was high glucose DMEM supplemented with 10% human serum (HS Lonza), 2 mM L-glutamine, 1% NEAA and 1% penicillin-streptomycin (Life Tech, Paisley, UK).

*Feeder cells*

Human dermal feeder (hDF) cells originated from neonatal foreskin fibroblasts were grown under GMP compliant conditions and donated by Intercytex Ltd. Two lines (P106090049 and P107080110) were selected for optimal growth and maintenance of hESC self renewal and pluripotency, and used for further derivation protocols. HDFs were cultured in DMEM +10% human serum (HS) for several passages using Trypzean (Sigma, Dorset, UK) to passage at a ratio of 1:3 and mitotically inactivated at p9-11 for a minimum of 5 hours using 10 μg/ml Mitomycin C (Sigma, Dorset, UK) at 37^o^C 5% CO_2_. All culture was performed to GMP standards within our clinical grade facility.

MEFs used for the expansion and characterisation of hESC lines, were derived from day 13.5 MF1xCD1 mouse embryos and the resulting fibroblast cells were cultured with 10% fetal bovine serum (FBS), 2mM L-glutamine and 1x penicillin-streptomycin (Life Tech, Paisley, UK). Cells were passaged at a ratio of 1:3 until passage 4 and then mitotically inactivated for 3h using 10ug/ml mitomycin C (Sigma, Dorset, UK). Prior to hESC addition, MEFs were plated at a density of 6x10^4^ cm^2^ on 0.1% gelatin coated plates 24 hours before use.

*Derivation of hESC lines*

**General procedure for inner cell mass (ICM) isolation and plating.** Blastocysts at day 5-7 were graded according to our modified grading system (above). The zona pellucida was removed by a brief treatment (<1 minute) with acid Tyrode’s solution (pH 2.5, Sigma-Aldrich). The denuded blastocysts were washed in hESC derivation media (as dictated by the derivation culture system), and transferred into a drop of the medium. The trophoblast cells were mechanically and manually disrupted with the aid of two pulled glass Pasteur pipettes, one with a cutting, open end and the other with a closed round end, or cut with Stem Cell Cutting Tool (Swemed Lab International AB, Billdal, Sweden) directly. The ICM-containing cell clump was gently triturated and transferred to a lawn of mitotically inactivated hDF feeder cells in an organ culture dish (OCD, Becton Dickinson). Alternatively for poor quality blastocysts without visible ICMs, the intact denuded embryos were directly transferred onto a lawn of mitotically inactivated hDF feeder cells in an OCD. The embryo was disrupted manually with the Stem Cell Cutting Tool within 10 days from fertilisation (day 0).

**Derivation culture systems.** The derivation culture systems are combinations of various culture media with different adhesive matrices, and hDF feeder cells (see Results). All cultures were performed at 37^o^C in 5% CO_2_. The primary colonies of hESCs were split by manual dissection to new feeder layers after 3–5 days for each passage until Passage 5-8, at which point some colonies were frozen. The cells were then passaged enzymatically with trypLE Select (Invitrogen). To cryo-preserve hESC lines, all cells were treated with trypLE Select for 4-5 minutes and were slow-frozen in 90% FBS or HS and 10% DMSO (D2650 Sigma- Aldrich) in a Nalgene® Mr Frosty (Sigma-Aldrich) at -80^o^C overnight before transferring into -196C in LN2. Masterbanks were created for each of the clinically derived stem cell lines MAN10 – MAN16 within our grade A GMP facilities. Morphologically good quality colonies were passaged onto fresh cultures of inactivated hDFs every 6-7 days in TeSR2, using the EZpassage tool (Life Tech, Paisley, UK) to maintain pluripotency and ensure low karyotypic abnormality build-up. Cells were passaged in this manner until passage 13-14 and then frozen down in bulk as 35-45 vials, either using 90% HS (Lonza) and 10% DMSO (Sigma, Dorset, UK) or using Profreeze (Lonza) a cGMP freezing solution supplemented with 15% DMSO. Cells were transferred immediately to -80^o^C, then stored after 24 hours under LN2 (vapour phase). All cells were screened for absence of mycoplasma contamination using both PCR and Elisa-based assays and characterised as below before being deposited in the UK Stem Cell Bank.

*Immunochemical staining of pluripotency markers*

Cell surface markers and transcription factors characteristic of pluripotent hESCs were detected using immunocytochemistry. The cells were fixed with 4% paraformaldehyde, and incubated with antibodies against stage specific embryonic antigens SSEA-4, SSEA-1, TRA-1–60, TRA-1-81 (Abcam), and transcription factors SOX2, NANOG (Cell signalling Technologies) and OCT-4 (BD Biosciences) at 4^o^C overnight. Secondary antibodies (Life Technologies) conjugated to FITC or TRITC were used to detect the presence of the markers using a BX51 microscope (Olympus, Hertfordshire, UK) equipped with a Q-Imaging camera (Micro Imaging Applications Group, Inc, Buckinghamshire, UK). Image processing was done with the aid of Q-Capture Pro software package (Micro Imaging Applications Group, Inc). A threshold of at least 70% positive cells was used as the limit for a positive result but the majority of stained colonies had >90%.

*Differentiation in vitro*

Undifferentiated hESC colonies were mechanically cut and cultured in suspension in medium FM for 10-14 d to form embryoid bodies. They were subsequently plated onto FBS-coated glass coverslips and cultured in the same medium for two weeks. After culture, cells were fixed in 4% paraformaldehyde, and immunofluorescence detection of common differentiation markers of the three germ layers- Ectoderm markers: Beta-Tubulin 3, neurofilament (R&D Systems); Endoderm markers: GATA6, FOXA2 (Cell Signalling Technologies); and Mesoderm markers Alpha-smooth muscle actin and Vimentin (R&D Systems) were performed to indicate ability to differentiate into all three germ layers. Secondary antibodies (Life Technologies) conjugated to FITC were used to detect the presence of the markers using a BX51 microscope (Olympus, Hertfordshire, UK) equipped with a Q-Imaging camera (Micro Imaging Applications Group, Inc, Buckinghamshire, UK). Image processing was done with the aid of Q-Capture Pro software package (Micro Imaging Applications Group, Inc).

*Teratomas*

Approx 3 x10^6^ hESCs were inoculated under the kidney capsule of 2 month old SCID mice. The animals were killed at 8 weeks and the tumour dissected and fixed in 4% paraformaldehyde and embedded in paraffin by iGENTBio (San Diego, USA). Sections were stained with Haematoxylin and Eosin or Alcian blue.

*Karyotyping*

MAN 10-16 hESC lines were karyotyped following a BrdU/colcemid harvest method, fixation with 3:1 methanol:acetic acid and G-banding. Mitotically active cells were treated overnight in media containing 400 mg/ml BrdU and 125 ng/ml colcemid. 16-18 hours later the cells were detached with Trypsin/Versene, swelled in a hypotonic solution (28mM potassium chloride, 0.4%w/v tri-sodium citrate) and fixed in an ice-cold 3:1 solution of methanol:acetic acid. The fixed cells were then spread onto slides and the chromosomes stained with Leishman’s staining solution. A minimum of 30 cells were counted with no mosaicism reported except for one single cell in MAN 12.  Images were obtained using Metasystems image capture and karyotyping software and are shown at approximately x630 magnification.

*CGH arrays*

Genomic DNA was isolated from feeder free hESC cultures using a phenol/chloroform DNA extraction method according to lab protocols. Triploidy and common aneuploidies were excluded, and the sex chromosome complement determined by QF-PCR using locally designed primers for microsatellite markers located on chromosomes 13, 18, 21, X and Y.

hESC DNA and sex matched, reference human genomic DNA (Promega) were labelled using a CytoSure™ HT Genomic DNA labelling kit (Oxford Gene Technology) according to manufacturer’s protocols, with Cy3 dCTP used for hESC DNA and Cy5 dCTP used for reference DNA. The labelled DNA samples were co-hybridised to CytoSure™ ISCAv2 8x60K Comparative Genomic Hybridisation (CGH) microarrays (Oxford Gene Technology) at 65°C for 22hours in a Mai Tai® hybridisation system (SciGene). The microarrays were then washed and scanned at 2µm resolution using the G2656A microarray scanner system (Agilent Technologies).

Data analysis was performed using CytoSure™ Interpret software (v4.3.2 or v4.4.6) (Oxford Gene Technology) according to local protocols. Briefly, quality metrics were assessed for suitability and the data visualised to exclude large mosaic aberrations. Aberrations were detected using automated settings with a minimum 4 probes and a mean log2 ratio threshold of 0.35 for gains and -0.6 for losses. Copy number aberrations with a population frequency of >1% in databases of normal variation [[6](#_ENREF_6)] were considered polymorphic variants and manually filtered.

*HLA Typing*

Human Leukocyte Antigen typing was performed at intermediate resolution for HLA-A, B and C loci and at high resolution for HLA-DRB1 and DQB1 using LABType^®^SSO kits (One Lambda, inc). All reagents were processed using a LABXpress^®^ robotic system, integrated with a Luminex^®^ Liquichip 200 analyser. Data analysis was performed using HLA Fusion^TM^ v3.0.

SUPPLEMENTAL REFERENCES

References

1. Camarasa MV, Kerr RW, Sneddon SF, Bates N, Shaw L, Oldershaw RA, Small F, Baxter MA, McKay TR, Brison DR, Kimber SJ: **Derivation of Man-1 and Man-2 research grade human embryonic stem cell lines.** *In Vitro Cellular & Developmental Biology-Animal* 2010, **46:**386-394.

2. Murdoch A, Braude P, Courtney A, Brison D, Hunt C, Lawford-Davies J, Moore H, Stacey G, Sethe S, Procurement Working Grp Natl C: **The Procurement of Cells for the Derivation of Human Embryonic Stem Cell Lines for Therapeutic Use: Recommendations for Good Practice.** *Stem Cell Reviews and Reports* 2012, **8:**91-99.

3. **UK National Clinical human Embryonic Stem Cell Forum** [<http://www.clinicalstemcellforum.org.uk>] Accessed 04 April, 2017.

4. Balaban B, Brison D, Calderon G, Catt J, Conaghan J, Cowan L, Ebner T, Gardner D, Hardarson T, Lundin K, et al: **Istanbul consensus workshop on embryo assessment: proceedings of an expert meeting.** *Reproductive Biomedicine Online* 2011, **22:**632-646.

5. Stephenson J: **In vitro fertilization.** *Jama-Journal of the American Medical Association* 2008, **299:**2737-2737.

6. **Database of Genomic Variants** [<http://dgv.tcag.ca/dgv/app/home>] Accessed 04 April, 2017.
