# Supplementary figures and images for: High quality clinical grade human embryonic stem cell lines derived from fresh discarded embryos
Source: Stem Cell Res Ther. 2017 Jun 5;8:128. doi: 10.1186/s13287-017-0561-y (PMC5460457; doi:10.1186/s13287-017-0561-y)

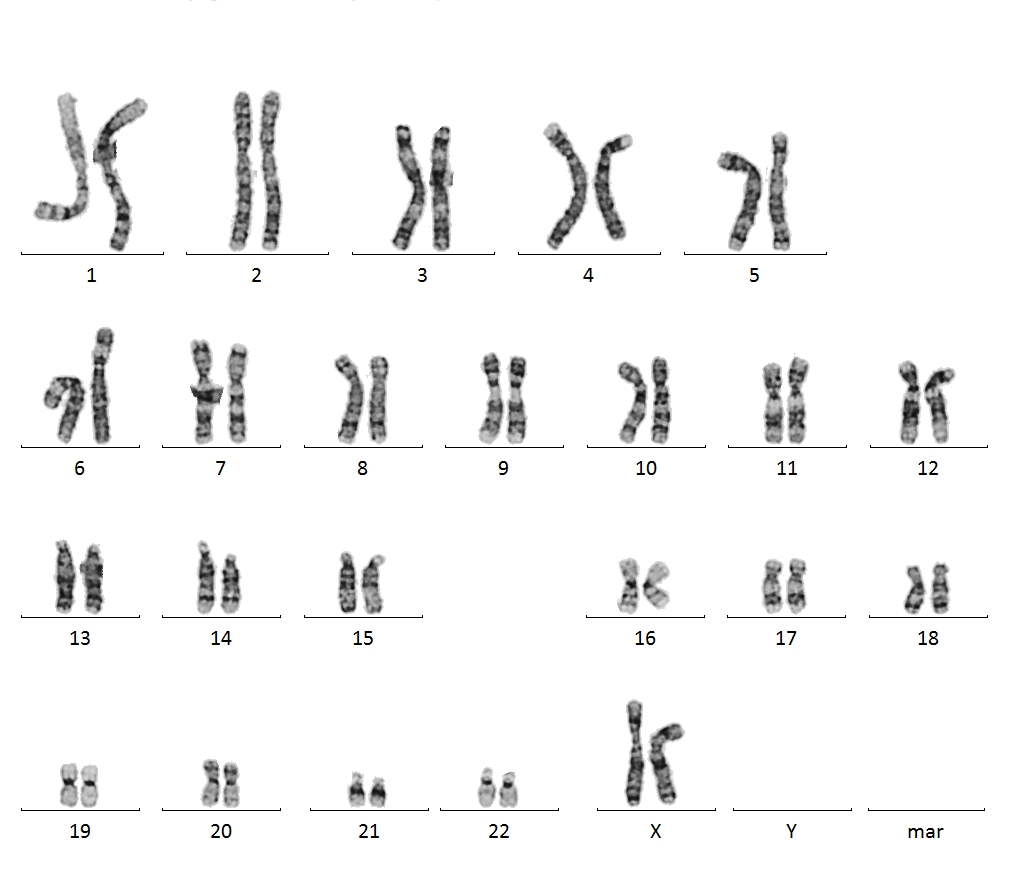

Supplement: Additional file 1: — Supplemental information includes supplemental experimental procedures, one figure and one table. (ZIP 292 kb) [file 13287_2017_561_MOESM1_ESM.zip › Figure S1a .JPG]

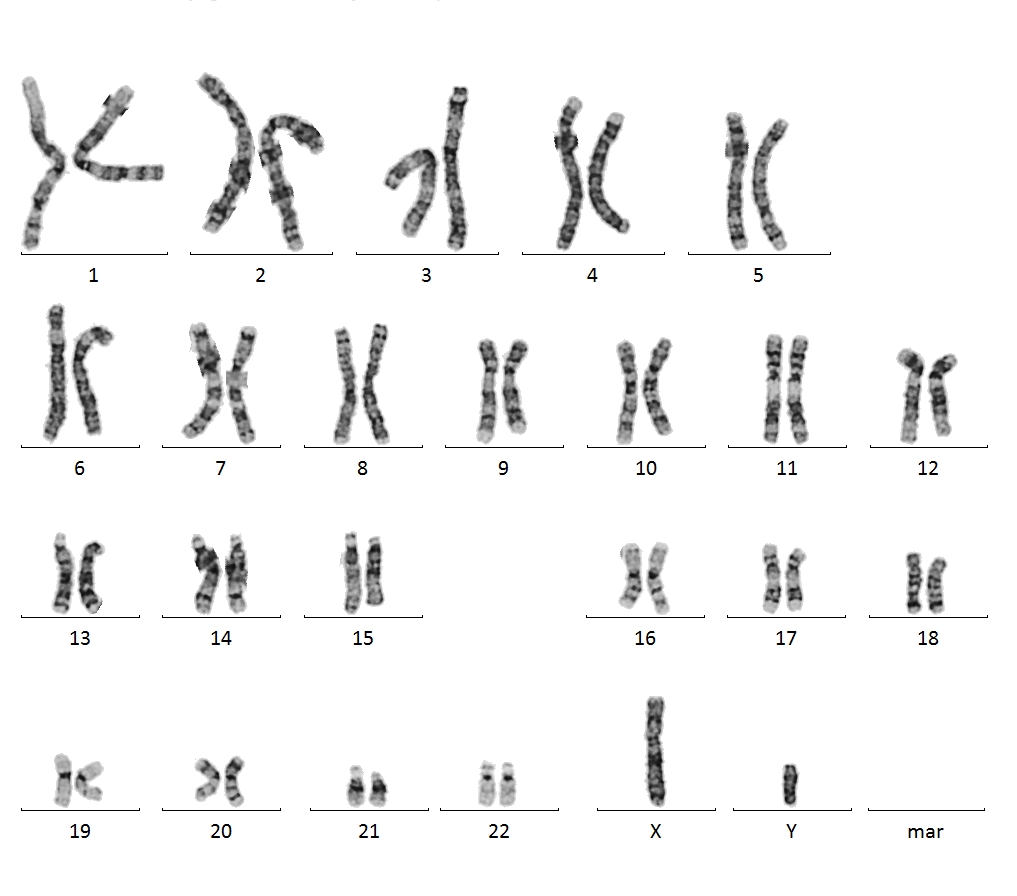

Supplement: Additional file 1: — Supplemental information includes supplemental experimental procedures, one figure and one table. (ZIP 292 kb) [file 13287_2017_561_MOESM1_ESM.zip › Figure S1b .JPG]
